# Knowledge Mobilization Efforts by Global Pediatric Oncology Civil Society Organizations: An Environmental Scan Protocol

**DOI:** 10.1177/30502225251383342

**Published:** 2025-10-29

**Authors:** Emily K. Drake, Angelina Lui, Ekaterini Damoulianos, Patrick Cossette, Michel Duval, Ramandeep S. Arora, Megan Doherty, Chiquita R. Hessels, YiQing Lü, Samiratou Ouedraogo, Dilek Sayik, Argerie Tsimicalis

**Affiliations:** 1 McGill University, Montréal, QC, Canada; 2 Leucan, Montréal, QC, Canada; 3 Centre Hospitalier Universitaire Sainte-Justine, Montréal, QC, Canada; 4 Départment de Pédiatrie,Université de Montréal, Montréal, QC, Canada; 5 Max Super Specialty Hospital, Saket, New Delhi, India; 6 Indian Pediatric Hematology Oncology Group (INPHOG), Gurugram, Haryana, India; 7 Department of Pediatrics, Faculty of Medicine, University of Ottawa, Ottawa, ON, Canada; 8 Two Worlds Cancer Collaboration, North Vancouver, BC, Canada; 9 Li-Fraumeni Syndrome Association of Canada, Nanaimo, BC, Canada; 10 Université Joseph KI-ZERBO, Ouagadougou, Burkina Faso; 11 Institut National de Santé Publique, Ouagadougou, Burkina Faso; 12 European University of Lefke, Northern Cyprus

**Keywords:** knowledge mobilization, cancer, children, teens, protocol, global health, global oncology, rare

## Abstract

Finding reliable, evidence-based information and resources concerning pediatric oncology can be a challenge for childhood cancer community members worldwide. The knowledge mobilization activities of civil society organizations around the world can improve the creation and sharing of pertinent informational resources. A comprehensive understanding of all of the civil society organizations that exist, and their knowledge mobilization activities is missing in the extant literature. This environmental scan of online resources will provide a snapshot of civil society organizations that serve the pediatric oncology community around the world and map the different ways that they disseminate information. Through mapping this organizational landscape, our study will highlight existing gaps and propose novel strategies. This novel global environmental scan methodology will outline each step acting as a guide for others seeking to optimize and magnify knowledge mobilization efforts.

## Introduction

Approximately 400 000 children are affected by cancer worldwide annually, with only half of these children ultimately receiving a diagnosis.^1^ Of those treated, 80% are cured in high-income countries compared to 30% of those living in low-middle income countries.^1^ Navigating economic barriers, healthcare systems, and comprehensive information, along with the physical and psychosocial challenges of a pediatric cancer diagnosis, is a challenge experienced by children and their families throughout the world.^2^ Within each country, the accessibility of reliable health information varies with culture, research, and healthcare systems.^3^ Civil society organizations that serve the pediatric oncology community may act as reservoirs of information that allow people to better understand these diseases, how to navigate diagnoses, and learn of treatments available.^4^ They can act as a knowledge broker by disseminating research findings and fostering collaborations^5^. Civil society organizations may also take action in unique ways through funding initiatives, creating awareness campaigns, pursuing advocacy efforts for policy changes, and directly supporting treatment programs and psychosocial services.^6^

Knowledge mobilization (KMb) is a novel term originating from the implementation of scientific research.^7^ The definition for KMb describes different activities that contribute to how knowledge is created and made accessible to the public, so that it can be implemented into public policy and professional practice.^8^ KMb is important in ensuring that knowledge is effectively utilized to create positive impacts in education, innovation, and community engagement, and mitigate knowledge inequalities.^7^ In pediatric oncology, as in healthcare in general, the use of increasing research evidence is often restricted by the limited uptake by patients, healthcare professionals, and industry workers.^9^ This knowledge gap is further amplified between countries based on differences in access to health care and health literacy.^10^ KMb aims to propel the adoption of novel interventions for better treatment outcomes.^11^ implement more evidence-based practices,^12^ and address the unique challenges experienced by childhood cancer survivors.^13^ China, the United States, and Turkey contributed the most pediatric oncology publications as of 2016, which suggested a correlation between research productivity and gross domestic product.^14^

Disparities in access to knowledge worldwide led the World Health Organization (WHO) and St. Jude Children’s® Research Hospital^15^ to launch the Global Initiative for Childhood Cancer.^16^ This initiative aims to sustain 60% survival rates for children with cancer globally, while reducing overall suffering. This initiative outlines the services available and survival rates; however, it lacks a comprehensive map of the civil society organizations available to the pediatric oncology community. In Canada, ACCESS^17^ was created to advance research and support the childhood cancer community in enhancing the well-being and experiences of children and their families. The extensive network lacks a comprehensive list of global civil society organizations that serve the pediatric oncology community worldwide.^18^ Civil society organizations available in each country have yet to be identified or outlined in a single source. Environmental scans are an effective tool for studying KMb activity through systematic gathering of data surrounding specific initiatives to map a comprehensive overview of the current knowledge dissemination landscape.^19^ Environmental scans are used in health research to facilitate decision-making in response to changing circumstances.^20^ An environmental scan summarizing this data will provide a better understanding of the characteristics of the civil society organizations that exist, how knowledge is disseminated in different countries, and provide insight on how we can connect organizations to unify efforts and maximize our voices together to drive meaningful and transformative changes.

### Objectives

This environmental scan will identify the knowledge mobilization efforts currently underway globally for the pediatric cancer community, by: (1) mapping a current list of civil society organizations that serve the pediatric oncology community around the world; (2) describing and analyzing the means through which these global cancer organizations disseminate information; (3) identifying gaps in global KMb efforts and potential opportunities to increase KMb capacity in pediatric oncology around the world; and (4) describing a methodology for conducting a global environmental scan that uses various internet-based sources, unique means of engaging the global pediatric oncology community, and novel member checking efforts.

## Methods

### Study Design

Our study team, comprised of global pediatric oncology community representatives (non-profit employees, trainees, people with lived experience, scientists, and healthcare professionals), will conduct an environmental scan to systematically map and analyze civil society organizations around the world that serve the pediatric oncology population and their knowledge dissemination activities. With limited guidance available pertaining to the use of environmental scan methodology for health research,^21-23^ we designed a novel environmental scan methodology to conduct a multi-modal search of the internet to meet our global study objectives.

The development of this protocol was completed alongside the development of a national environmental scan protocol^24^ with a bilingual search strategy that has been uniquely designed to map the non-profit organizations that serve the pediatric oncology community in Canada and their KMb activities. Thus, this global protocol will not concern organizational activity in Canada. The development of this study protocol was also guided by the environmental scan methods of Castro et al,^21^ Choo,^23^ and Rowel et al,^22^ as well as updated methodological guidance for the conduct of scoping reviews.^25^ This environmental scan will be comprised of (1) conducting a comprehensive search of the websites and resource lists of global organizations; (2) screening search results with study inclusion criteria; (3) extracting relevant data; (4) conducting consultations with pediatric oncology experts; (5) analyzing extracted data; and (6) producing a final manuscript.^21^

### Eligibility Criteria

Civil society organizations will be eligible for inclusion in the environmental scan if they: have a registered charity number on their website or describe themselves as charitable organizations, civil society organizations, not-for-profits or non-profits. Through developing our national environmental scan protocol,^24^ we learned the terms non-profits, not-for-profits and charitable organizations used by the community differ from the government. In the same essence, different governments throughout the world may define these terms differently than the Canadian government. In essence, our global environmental scan will include organizations that list themselves as 1 of these 4 terms. In consultation with our team, we determined that the term civil society organization^26^ which describes organizations that are not businesses or for-profit associations, is better representative of the essence of organizations that we are seeking to identify around the world. Throughout this text, we will collectively refer to these organizations as civil society organizations for simplicity. To be included in the review, these organizations must be: (1) located outside of Canada (a separate review is being conducted by our team for that country)^24^; (2) focused on global, national or regional issues; (3) disseminating information to the public; and (4) serving the pediatric oncology community (e.g., researchers, siblings, patients, decision-makers) through a focus on 1 specific childhood cancer or for the broader childhood cancer community. Organizations will be excluded if their mission focuses on the broader cancer community (e.g., organizations that serve people of all ages who are diagnosed with cancer), the broader child health community (e.g., developmental disabilities) or the adolescent and young adult (ages 15-39 years of age)^27^ cancer community with a focus on those solely over the age of 18.

Knowledge mobilization activities pertaining to dissemination will be screened through this study to map and describe global efforts underway in this area by civil society organizations. These organizational activities will be eligible for inclusion in our environmental scan if they: (1) pertain to the dissemination of knowledge (e.g., online offerings, posts advertising in person activities); (2) are specific to pediatric oncology; and (3) concern oncology-relevant information (e.g., prevention, bereavement, end-of-life, treatment, psychosocial issues, survivorship, diagnosis).

### Data Sources and Search Strategy

#### Internet-Based Search of the Knowledge Dissemination Activities of Global Pediatric Oncology Civil Society Organizations

A 4-step quadrilingual search strategy will be employed to identify civil society organizations that serve the pediatric oncology community around the world. These steps will involve identifying civil society organizations by: (1) searching the civil society organizations that are a part of large global pediatric oncology organizations,^28^ (2) searching global civil society organizations that may be linked with ACCESS,^17^ and (3) searching the websites and resource lists/recommended links of pediatric oncology organizations on every continent. Our search strategies were developed in parallel with the development of our Canadian environmental scan^24^ by our study team and were reviewed by all consulting authors.^21^ Our mission was to develop a search strategy that is reflective of how childhood cancer community members (e.g., friends of a childhood cancer survivor, not-for-profit organization, researchers) would search for knowledge from organizations and/or partner with organizations to co-create knowledge dissemination products/strategies. The search strategy is guided by 12 keywords/terms (e.g., pediatric cancer, AYA; see Table 1 for the full list) used around the world that have been translated from English to French, Korean and Greek, based on the language abilities of our multi-stakeholder team. Civil society organizations in *Step 1* were purposively chosen to represent and guide our search on each continent through means feasible for our research team, representing countries of different income categorizations. Websites that are outside of our quadrilingual language capabilities will be translated.

**Table 1.** Key Search Terms for Global Strategy English Keywords Related to the Study Objectives Were Translated to French, Korean and Greek by Multilingual Study Co-authors (E.D., A.L.).

Original English keywords	Translated French keywords	Translated Korean keywords	Translated Greek keywords
Childhood cancer	Cancer chez l’enfant	어린시절 암	ΠαιδιΚός ΚαρΚίνος
Pediatric cancer	Cancer pédiatrique	소아암	ΠαιδιατριΚός ΚαρΚίνος
Paediatric cancer	Cancer pédiatrique	소아암	ΠαιδιατριΚός ΚαρΚίνος
Pediatric oncology	Oncologie pédiatrique	소아 종양학	ΠαιδιατριΚή ογΚολογία/ΠαιδοογΚολογία
Paediatric oncology	Oncologie pédiatrique	소아 종양학	ΠαιδιατριΚή ογΚολογία/ΠαιδοογΚολογία
Paediatric	Pédiatrique	소아	ΠαιδιατριΚό
Pediatric	Pédiatrique	소아	ΠαιδιατριΚό
Child cancer	Cancer de l’enfant	어린이 암	ΠαιδιΚός ΚαρΚίνος
Adolescent cancer	Cancer de l’adolescent	정소년 암	ΕφηβιΚός ΚαρΚίνος
Teen cancer	Cancer chez les ados	십대 암	ΚαρΚίνος Εφήβων
AYA	AJA	No direct translation	No direct translation
Infant cancer	Cancer du nourrisson	유아암	ΚαρΚίνο σΕ βρέφη

### Step 1: Searching Online Resources and Databases

#### Identifying the Partners of Major Global Initiatives

The first step will be to search the International Society of Paediatric Oncology’s website (SIOP)^29^ for civil society organizations that are listed/linked to this global membership. SIOP^28^ is a global multidisciplinary society whose sole focus is on pediatric and adolescent oncology. Our search of this website will include compiling those listed in the “education” section under “links and downloads,” and their 3 affiliated societies (The Pediatric Radiation Oncology Society’s resource list, The International Society of Paediatric Surgical Oncology’s affiliated societies, Childhood Cancer International’s member organizations). Next, the World Health Organization’s Global Initiative for Childhood Cancer’s^16^ portion of the World Health Organization’s website will be searched comprehensively to see if there are any listed civil society organizational partners throughout the text and listed resources.

#### Identifying ACCESS’ Global Civil Society Organizational Partners

We will search the civil society organizations that have been identified through the Canadian Institutes of Health Research’s 2022 Team Grant opportunity for the creation of a pediatric cancer consortium; those identified in the submission for the creation of ACCESS and current identified partnerships of the ACCESS network.^30^

#### Search the Resources and Directories of Organizations on Each Continent

The following organization’s websites will be searched along with their resources or lists of useful links for recommended civil society organizations, if applicable. These organizations were identified and selected as starting points based on the familiarity of the research team with civil society organizations in other countries and continents. For Asia, we will search Indian Pediatric Hematology Oncology Group (INPHOG),^31^ CanKids KidsCan India^32^ and Korea Childhood Leukemia Foundation.^33^ North American organizations that will be searched include ACCESS^17^ and St. Jude Children’s® Research Hospital.^15^ Australian organizations will include Leila Rose^34^ and Canteen.^35^ Our search of African civil society organizations will include CHOC Childhood Cancer Foundation South Africa^36^ and Children’s Cancer Hospital Foundation – Egypt.^37^ Our South American search will explore Fundación Ayúdame A Vivir^38^ in El Savador and Projeto Dodoi^39^ in Brazil. In Europe, we will search la Société Française de lutte contre les Cancers et les leucémies de l’Enfant et de l’adolescent^40^ in France and Floga in Greece.^41^

#### Step 2: Screening

A preliminary search of the results listed in resource directories was conducted to determine feasibility of the search strategy based on our team’s resources. An Excel spreadsheet will be used to manage the search strategy results, including the removal of duplicate items. The websites of each civil society organization that is retrieved through the search strategy will be assessed for study eligibility. Civil society organizations that do not meet the study inclusion criteria will be excluded from the environmental scan. Where questions arise around study inclusion and eligibility may be unclear, discussions will be held with members of the team to achieve a conclusion.^21^ Google translate^42^ will be used for resources that are published in languages other than those included in our quadrilingual search strategy.

#### Step 3: Data Collection

After search strategy results are screened for study eligibility, the websites of civil society organizations that meet study inclusion criteria will be listed in our drafted data extraction form (see Table 2). This template was developed with guidance from the work of Cooper et al.,^43^ to help standardize the process of data extraction. Our intention is to have this data extraction form serve as a working document where categories may be inductively added based on the dissemination strategies that are identified through the results of our search strategy. Team meetings will be had to discuss the potential addition of new categories and any uncertainties that arise in the data extraction process.^21^

**Table 2.** Data Extraction Template.

Organization	Continent	Country	Year founded	Cancer type (all or specific cancers)	Audience(s)	Language(s)	Social media Profiles (X, LinkedIn, Facebook, Snapchat, Instagram, Threads, Bluesky, TikTok, YouTube)	Videos	Blog	Pamphlet	Poster	Clinical practice guidelines	Online training (webinars)	In person training	Policy briefs	Academic publications
																
																
																

Data extraction will be conducted as a collaborative team effort and a reviewer will extract information pertaining to the civil society organization’s listed dissemination activities and organizational characteristics. Defining characteristics that our study will capture include where the organization is located (e.g., continent, country), the founding year, the language(s) of their content and their websites’ targeted audiences (e.g., researchers, siblings, children; based on how sections of their website are labeled). Data pertaining to their knowledge dissemination strategies (e.g., webinars, social media channels, reports) will be deductively extracted.

#### Step 4: Conducting Expert Consultation

Expert consultations will be conducted throughout the conduct of this study to strengthen the ability of this environmental scan to capture the global pediatric oncology civil society organizations and their knowledge dissemination activities in a manner that is based on our available resources (e.g., trainee time). Members of the ACCESS^17^ pediatric oncology network in Canada, pediatric oncology partners, digital opinion leaders and other organizations, will be presented with lists of preliminary results from our search strategy and asked to provide any other organizations they believe could meet our study inclusion criteria and that should be added to our list to screen.^21,44^ Our research team includes global members of the pediatric oncology community from different continents who will share familiar organizations and extract data. Preliminary results will be shared through posts on social media channels (e.g., LinkedIn, Bluesky, Instagram, X) by members of the research team, to ask the public and broader oncology community to review the list and provide further suggestions for consideration.^21^

#### Step 5: Data Presentation

Data generated during the screening process will be presented in a Preferred Reporting Items for Systematic Reviews and Meta-Analyses (PRISMA) flow diagram.^45^ The resultant manuscript will include a narrative summary that describes to the reader how the results of the environmental scan address the study objectives.^46^ Data extracted from the websites of the included global civil society organizations will accompany this narrative in graphical forms that best present the collected data and explain how it relates to study objectives. We anticipate that these will include graphical (e.g., bubble charts, bar charts, graphic images) and tabular images that capture both the multitude of ways that organizations disseminate information around the world and also, the characteristics of the organizations themselves (see Table 3 for an example).^46^

**Table 3.** Data Presentation Example.

Civil society organization characteristic	Results
Continent	# by continent
Country	# by country
Year founded	# by year founded
Language(s) of content	# by languages
Audience for content	# by audience
Cancer type	# by cancer type

### Patient and Public Involvement

One of the study’s co-authors (BLINDED) is a person with lived experience. Five of our co-authors assisted with the preparation of the manuscript and are affiliated with civil society organizations (BLINDED).

## Results

Expert consultations will be conducted throughout the winter and spring of 2025. Pending this milestone, it is anticipated that the final results from the study will be available by the summer of 2025.

## Discussion

This environmental scan protocol outlines the steps of a global environmental scan that will map civil society organizations around the globe and describe the means through which they disseminate knowledge. By doing so, we will be able to identify their countries, languages, audiences, and subsequently identify gaps that exist in this area of KMb. The steps chosen to guide this environmental scan search strategy were done so through a lens of feasibility based on our team’s resources. We recognize that a comprehensive search of every country and in additional languages outside of the expertise of our team, are outside of the means we have available. It is thus our aim to provide an overarching review of some of the major pediatric oncology civil society organizations around the world and to make our protocol, study materials, and resulting manuscript accessible for others to leverage our novel environmental scan methodology and tailor it to their local context (e.g., CanKids KidsCan, the national society for change for childhood cancer in India).^32^

### Dissemination

Key partners were recruited to partake in the development of our protocol and conduct of the environmental scan. These team members will be involved with disseminating the findings resultant of this review to their communities throughout the world. This work is part of a broader KMb project that is identifying the needs of unique stakeholders. Along with a Canadian environmental scan,^24^ these results will inform the strategies that we develop for our Canadian pediatric oncology consortium, ACCESS.^17^ To maximize research impact beyond the Consortium, our group is also to developing and mobilizing resources globally. These study results will help inform the priorities of the strategic KMb plan we develop alongside plans to make such efforts sustainable. In particular, understanding the audiences, locations, languages, and dissemination strategies, will shed light on where gaps remain when considering global cancer resources, education, and support. We anticipate that findings from these 2 environmental scans will allow us to develop tools, education products (e.g., videos, infographics), guidelines, and strategies to be implemented for national and global audiences. In addition to these, we plan to present the results through traditional academic forums including academic presentations and open access publications.

## Conclusion

Civil society organizations are integral to disseminating evidence-based information to communities and academics. However, there are limited understandings of who these global organizations are and how they share information. This protocol was developed to guide an environmental scan that will map the civil society organizations that exist around the world to serve the pediatric oncology community and the means through which they disseminate information, to address the aforementioned research objectives. By doing so, we will be able to highlight areas of opportunity for global oncology KMb and gaps that exist. Through outlining steps in our methodology, others will be able to utilize this protocol as a guide to develop and conduct their own environmental scans.
